# Psychiatry Boot Camp Podcast: Psychiatric Fundamentals for Clinical Learners

**DOI:** 10.1007/s40596-024-01979-7

**Published:** 2024-05-09

**Authors:** Mark Mullen, Hesham Essa, Brooke Gertz, Sara Bharwani, Jyotsna Ranga, Donna Sudak

**Affiliations:** 1 Creighton University, Omaha, NE USA; 2 Drexel University, Philadelphia, PA USA

A recent study of first- and second-year residents found that respondents unanimously cited “residency preparation courses,” often called boot camps, as crucial to support the transition to residency [1]. These courses exist both in medical school prior to graduation and in residency programs. Although the efficacy of these courses has not been robustly investigated, participation in them has been associated with increased learner-perceived preparedness for residency [2, 3]. Participation in such courses has also been associated with increases in skills, knowledge, and confidence for learners near the transition to residency [4]. Institutionally created “boot camps” require considerable faculty time and resources from the institutions, are limited to a course’s temporal boundaries, and require in-person attendance from learners. Virtual “boot camps” are one solution to these barriers, but still require significant institutional commitment. We attempted to make the content of “boot camps” more scalable, globally available, and not limited to the timeframe or location of a particular course by translating the content into a podcast.

Podcasting is rising in popularity throughout medical education [5], with psychiatric learners appreciating the practicality and conversational tone [6]. Podcasting facilitates adult learning [7] and aligns with the ongoing movement away from lectures and toward active learning models that spur classroom discussion [8]. The podcast *Psychiatry Boot Camp* was designed as a cohesive course to facilitate the transition to residency.

After releasing *Psychiatry Boot Camp*, we surveyed listeners to evaluate how the innovation is being used and how it could be improved. We also aimed to understand the podcast’s impact on professional skills and professional identity formation. Our hypothesis was that learners would find this form of education helpful. This article will discuss the podcast innovation, learner feedback received, and lessons learned so that psychiatric educators will recognize the promise of this method of engaging learners.

## Creating the Innovation

We reviewed the literature for psychiatric residency preparation courses and found no model curriculum. We designed a nine-episode podcast series based on existing literature about psychiatry residency preparation courses [9, 10], “psychiatry residency crash course” curricula at our home institution, and the clinical experiences of the creator (MM, a third-year psychiatry resident at the time of inception). Topics included the following (in order): introduction to *The Diagnostic and Statistical Manual of Mental Disorders*, clinical interviewing, mental status examination, suicide risk assessment, management of acute agitation, case formulation, decisional capacity, introduction to psychotherapy, and violence risk assessment. We explicitly tailored the content to fourth-year medical students transitioning into psychiatry residency.

Support was obtained for protected time from our department for the podcast creator. Funding for recording hardware, editing software, hosting software, artwork, and expert faculty honoraria was secured through a grant from the Behavioral Health Education Center of Nebraska. After familiarizing ourselves with the technical aspects of podcasting and securing an expert technical advisor, we set out to recruit expert faculty. Initially, we intended to provide an honorarium ($100), but after several declined, we provided “thank you” gifts instead.

We developed episode outlines based on publications or presentations from our experts, journal articles, textbook chapters, and web-based content. The outlines were sent to experts to be interviewed for review. Recordings were made via Zoom and lasted from 30 to 120 min based on the complexity of the topic and availability of the expert. Recordings were edited and posted to podcast directories freely available to learners across the globe. The first episode was released on February 15, 2023, with successive episodes released weekly so that the entire series was made available by May 2023. As of March 27, 2024, there have been 48,016 episode downloads from the nine-episode series.

The nine-episode series is now *Psychiatry Boot Camp* “Season 1.” Season 2, which we began to release in November 2023, is designed to impart principles of psychiatric diagnosis and is not included in our current investigation. We disseminated the podcast through the creator’s personal social media accounts, flyers around our home institution’s training sites, and word of mouth.

After releasing the podcast, we recruited respondents via the podcast feed and social media using the creator’s personal account. We emailed a brief, anonymous survey to 66 listeners, and 61 responded (92%). The Creighton University Institutional Review Board deemed this study as exempt.

## Learner Feedback

We excluded 18 responses due to empty or incomplete submissions, yielding 43 completed responses. Participants varied in their levels of training and practice. Most responses came from post-graduate year (PGY) 1 residents (*n* = 10), medical students in their clinical years (*n* = 8), advanced practice providers (nurse practitioner/physician assistant) (*n* = 6), and psychiatry attendings (*n* = 5). In smaller numbers, we received responses from PGY-2/3 residents, psychoanalytic trainees, medical graduates, and researchers. The most frequently accessed episodes in our survey sample were “Clinical Interviewing” and “Mental Status Examination,” with 35 each.

Listeners were asked to assess their podcast experience using a 5-point Likert scale (1 = strongly disagree, 5 = strongly agree), and the responses were averaged (Table 1). We did not detect any significant differences in responses between these groups.

**Table 1:** Listener feedback from survey items

	Average Likert score (95% confidence interval)
After listening to psychiatry boot camp	
I was more confident in my patient encounters	4.11 (3.85 to 4.37)
I was more confident in my knowledge of psychiatric evidence	4.19 (3.97 to 4.41)
I understood my patients more deeply	4.12 (3.83 to 4.4)
I found my work to be more meaningful	4.09 (3.82 to 4.37)
I was stimulated to research an episode topic further	4.16 (3.84 to 4.48)
I was more prepared to teach my peers	3.98 (3.7 to 4.25)
I had a better understanding of my own professional identity	4.09 (3.81 to 4.38)

1 = strongly disagree, 5 = strongly agree

In addition to the survey questions in Table 1, participants were prompted to rank textbook chapters, podcasts, journal articles, and formal didactic sessions from most to least helpful for overall learning and professional development. Formal didactic sessions were ranked highest, with an average rank of 1.95 (95% CI 1.62 to 2.28), but podcasts followed closely with an average rank of 1.98 (95% CI 1.7 to 2.25). Journal articles had an average rank of 2.81 (95% CI 2.55 to 3.08), and textbook chapters had an average rank of 3.23 (95% CI 2.92 to 3.54).

Respondents also received four open-ended questions about the podcast: what did you most gain from listening to *Psychiatry Boot Camp*; overall, what was particularly useful about *Psychiatry Boot Camp*; do you have any critiques of *Psychiatry Boot Camp*; and do you have any suggestions for future directions for *Psychiatry Boot Camp*. One author (BG) independently analyzed responses for themes. The two strongest themes regarding what was most gained from the podcast were that it provided pragmatic tips/skills for practice (especially pertaining to risk assessment and the mental status exam) and that it presented an overview of foundational psychiatric knowledge. The next most common theme was that listeners gained a sense of mentorship and insight from experts. Another frequent theme was that listeners acquired perspective about what it is like to be a psychiatrist. Four responders expressed that the podcast renewed their passion for psychiatry. Another four responders indicated that they learned about “unspoken rules” and gained psychiatric insight that is not taught in medical school or intern year of residency.

A significant theme regarding what was particularly useful about the podcast was that it felt “authentic” due to unscripted conversation and the infusion of personal experiences. Listeners also frequently mentioned they appreciated that the interviews were conducted by a resident. Many listeners particularly valued the ease of listening to the podcast and described it as “engaging,” “digestible,” and “entertaining.” Feedback was positive about the podcast interviewer, highlighting the value of a single interviewer throughout the podcast series to provide a cohesive narrative and a consistent voice—the voice of a “trusted friend.”

The majority of survey responders indicated that they had no critiques of the podcast. The most common critiques were that there were not enough episodes, and that some episodes were too long. Several respondents wanted interviewees with less expertise who may be more relatable to them, such as residents. Several respondents indicated a desire for more conversation between the interviewer and interviewee and to limit the duration of each episode. Many responders wanted supplemental material such as an accompanying online resource, quiz, or summary page. Suggestions for future directions of the podcast primarily included requests for particular topics. The only unique future suggestion was to design a podcast targeting the transition into the PGY-3 outpatient year of psychiatry residency.

## Lessons Learned

We aimed for a conversational, authentic interview style and avoided some common educational practices such as explicitly numbered learning objectives or assessment questions. This may have contributed to feedback about entertaining, engaging, and digestible content. Feedback requesting shorter episode durations and more conversation in *Psychiatry Boot Camp* episodes aligns with existing literature about another psychiatry podcast, *PsychEd*, where survey respondents appreciated “conversational” tone and 30–60-min episode duration [6].

The podcast clearly accomplished its goals to improve learner confidence, curiosity, passion, knowledge, and sense of purpose as these pertain to a career in psychiatry. Interpretation of the data is limited by the number of respondents and their educational diversity. The greatest limitation of the outcome is the selection bias inherent in assessing learning style and feedback from participants who chose to listen to *Psychiatry Boot Camp* and engage in the survey. Another significant limitation of our investigation was the population that responded to our survey; the podcast was explicitly developed for psychiatric learners navigating the transition to residency, but only 18 respondents (42%) could be considered a part of this demographic. This surprised us, and reflects the foundational nature of content taught in residency preparation courses. While the “voice” of the podcast speaks directly to learners navigating the transition, a wide range of professionals found the podcast to be helpful. Additionally, we did not ask if advanced practice providers were still in training or currently in practice, which limits our ability to interpret the data.

## Discussion

The number of podcast episode downloads (> 48,000) strongly attests to the demand for and reach of this learning modality. The averaged Likert scale ratings from our survey responders fell between agree and strongly agree for all outcomes except for increased confidence in teaching peers; the average Likert rating for this question fell between neutral and agree. One explanation for this neutrality may be the intended audience and group of responders; medical students and first-year residents are often “junior” members of clinical teams, and may not imagine themselves easily teaching other members of the team.

While the intent of this innovation was to fill an empty niche in psychiatric education by creating a podcast with a coherent narrative and specific purpose for a neglected audience (soon-to-be residents), we believe the data indicates it accomplished more than just dispersion of the usual knowledge base characteristic of educational “boot camps.” One listener stated that they gained “a conversation and a mentor” from the podcast, and another listener reported that it “made me excited to be a psychiatrist.” Another responder captured the spirit of this innovation by commenting that the podcast bridged the “large difference between being a good student and learning to be a professional.” Although the place for podcasts in medical education has yet to be fully established, this study indicates that podcasts are appreciated by various learners and can meaningfully contribute to education.

## Data Availability

Data is available upon reasonable request.
